# Circular RNA-related CeRNA network and prognostic signature for patients with oral squamous cell carcinoma

**DOI:** 10.3389/fphar.2022.949713

**Published:** 2022-12-01

**Authors:** Yaodong He, Dengcheng Yang, Yunshan Li, Junwei Xiang, Liecheng Wang, Yuanyin Wang

**Affiliations:** ^1^ Key Laboratory of Oral Diseases Research of Anhui Province, College and Hospital of Stomatology, Anhui Medical University, Hefei, China; ^2^ Department of Physiology, School of Basic Medical Sciences, Anhui Medical University, Hefei, China

**Keywords:** oral squamous cell carcinoma, high-throughput sequencing, circular RNA, ceRNA network, prognostic model

## Abstract

**Background:** Circular RNA (circRNA) has an important influence on oral squamous cell carcinoma (OSCC) progression as competing endogenous RNAs (ceRNAs). However, the link between ceRNAs and the OSCC immune microenvironment is unknown. The research aimed to find circRNAs implicated in OSCC carcinogenesis and progression and build a circRNA-based ceRNA network to create a reliable OSCC risk prediction model.

**Methods:** The expression profiles of circRNA in OSCC tumors and normal tissues were assessed through RNA sequencing. From the TCGA database, clinicopathological data and expression patterns of microRNAs (miRNAs) and mRNAs were obtained. A network of circRNA-miRNA-mRNA ceRNA was prepared according to these differentially expressed RNAs and was analyzed through functional enrichment. Subsequently, based on the mRNA in the ceRNA network, the influence of the model on prognosis was then evaluated using a risk prediction model. Finally, considering survival, tumor-infiltrating immune cells (TICs), clinicopathological features, immunosuppressive molecules, and chemotherapy efficacy were analyzed.

**Results:** Eleven differentially expressed circRNAs were found in cancer tissues relative to healthy tissues. We established a network of circRNA-miRNA-mRNA ceRNA, and the ceRNA network includes 123 mRNAs, six miRNAs, and four circRNAs. By the assessment of Genomes pathway and Kyoto Encyclopedia of Genes, it is found that in the cellular senescence, PI3K-AKT and mTOR signaling pathway mRNAs were mainly enrichment. An immune-related signature was created utilizing seven immune-related genes in the ceRNA network after univariate and multivariate analysis. The receiver operating characteristic of the nomogram exhibited satisfactory accuracy and predictive potential. According to a Kaplan-Meier analysis, the high-risk group’s survival rate was signally lower than the group with low-risk. In addition, risk models were linked to clinicopathological characteristics, TICs, immune checkpoints, and antitumor drug susceptibility.

**Conclusion:** The profiles of circRNAs expression of OSCC tissues differ significantly from normal tissues. Our study established a circRNA-associated ceRNA network associated with OSCC and identified essential prognostic genes. Furthermore, our proposed immune-based signature aims to help research OSCC etiology, prognostic marker screening, and immune response evaluation.

## Introduction

Oral squamous cell carcinoma (OSCC) is a group of malignant diseases that develop on the surfaces of the lips, gums, tongue, cheeks, and palate (Wong and Wiesenfeld, 2018). In 2020, the International Agency for Research on Cancer (IARC) reported that there were approximately 3,77,713 new cases of lip and oral cancer worldwide, accounting for 2.1% of all cancers (Kalogirou et al., 2021). OSCC is three times more common in men than women, and the average age of diagnosis is 50, although it also occurs in younger populations (Conway et al., 2018; Mello et al., 2018; Jehn et al., 2019). The occurrence and development of OSCC result from a combination of factors. Its main risk factors include tobacco and alcohol consumption and other possible risk factors, such as chronic irritation, poor oral hygiene, human papillomavirus (HPV), poor nutrition, and suppressed immune system (Petersen, 2009; Oji and Chukwuneke, 2012; Spence et al., 2016). These risk factors cause various genetic instabilities and molecular alterations, including loss of heterozygosity on chromosomes 3, 4, 7, 8, 11, 17, and 19, and downregulation of tumor suppressor genes such as TP53, RB, CDKN2A, and cancer upregulation of genes such as cyclin D1 (Leemans et al., 2011; Schaal and Chellappan, 2014; Alsahafi et al., 2019). Over the past few decades, there has been considerable progress in comprehensive treatment modalities for OSCC, with current treatments including radical surgery, radiation therapy, and chemotherapy (Oosting and Haddad, 2019). However, due to its late detection and high recurrence rate, mortality remains high, and the prognosis is relatively poor (Chang et al., 2021). Therefore, it is essential to explore the molecular mechanism of OSCC and the development of its malignant biological behavior and find effective targets for OSCC treatment.

At present, factors such as immune system disorders and tumor microenvironment (TME) have been confirmed to affect tumors’ occurrence, development, invasion, and drug resistance (Wu and Dai., 2017; Hinshaw and Shevde, 2019). The TME consists of tumor cells, tumor-associated stromal cells, and the extracellular matrix (Gao et al., 2019). TME can enhance or inhibit therapeutic effects and may have variable activation states (Vuong et al., 2019). Modifying or regulating specific factors or cells in the TME is particularly beneficial for treating tumors, such as immune checkpoint inhibitors (ICIs) (Liu et al., 2017). However, the distribution and mechanism of action of complex TMEs in OSCC have not been elucidated. In addition, it is necessary to explore methods further to accurately predict the efficacy of OSCC immunotherapy and find immunotherapy-related markers.

The circular RNA (circRNA) genome was discovered in a virus in 1976, but its role in gene regulation and cancer formation has only recently been discovered (Li et al., 2015). CircRNAs are a new type of non-protein-coding RNAs that are single-stranded with the head 3′ and tail 5′ ends covalently bound together to form a circular form (Geng et al., 2018). Due to the special covalently closed circular molecular structure, circRNAs are more resistant to degradation and more stable than traditional linear RNAs (Chen et al., 2016). In addition, circRNAs are suggested to be the universal molecules distributed in human cells; and in some circumstances, circRNAs are way more abundant than their linear isoforms. Many studies have shown that circRNAs can competitively adsorb miRNAs, thereby regulating gene expression at the post-transcriptional level (Gao et al., 2016). The circRNA hsa_circ_0009128 has been reported to be associated with the malignant progression of OSCC by targeting MMP9 to activate epithelial-mesenchymal transition (EMT) to stimulate OSCC cell proliferation and migration (Zhang et al., 2021). Cui et al. (2021) demonstrated that CircCDR1as elevates SLC7A11 as an oncogene for OSCC progression by targeting miR-876-5p. Shi et al. (2021) discovered that circGDI2 is a tumor suppressor that plays a role in OSCC pathogenesis by regulating FOXF2 expression through miR-454-3p. Furthermore, studies have shown that key nodal factors in the ceRNA network affect the proportion of tumor infiltrating immune cells (TICs) in the TME and the efficacy of immunotherapy. Wang et al. (2020) found that circRNA-002178 induced PD1 expression by sponging miR-34, thereby inducing T cell depletion. Ou et al. (2019) showed that circ_0000977 promoted the accumulation of hypoxia-inducible factor 1-alpha (HI1FA), inhibited NK cell lysis and led to immune escape of pancreatic cancer cells. However, there are few studies on the immune infiltration patterns of ceRNA and OSCC. At present, most of these studies use lncRNAs as the starting point to establish ceRNA networks and perform immune infiltration analysis. There is still a lack of related research on circRNAs.

In this study, we analyzed differentially expressed genes, including circRNAs, miRNAs, and mRNAs, based on high-throughput sequencing and the TCGA database. Public databases were used to examine interactions between circRNAs, miRNAs, and mRNAs, and to construct ceRNA networks. Genes within the ceRNA network were profiled by Gene Ontology (GO) and Kyoto Encyclopedia of Genes and Genomes (KEGG) pathway analysis. A prognostic mRNA signature associated with OSCC was established using univariate, Least Absolute Shrinkage and Selection Operator (LASSO) and multivariate Cox proportional hazards regression analysis and validated using time-dependent Receiver Operating Characteristic (ROC) curve analysis. Finally, we also assessed the association of this risk model with tumor-infiltrating immune cells or immune-related molecules, and the relationship of this model with the efficacy of chemotherapy in OSCC. Our study will contribute to a better understanding of the regulatory ceRNA network and provide a reliable reference for developing therapeutic targets for OSCC.

## Materials and methods

### Patient tissue samples and cell lines

For high-throughput sequencing, three pairs of quickly frozen OSCC tissue and surrounding healthy tissue were obtained from patients through the operation. Then, twenty pairs of samples were used for circRNA validation by quantitative real-time polymerase chain reaction (qRT-PCR), including samples for high-throughput sequencing analysis. Before the procedure, all experimental patient samples received no other therapies, and a thorough pathologic analysis validated all oral squamous cell carcinoma tissues. Each patient signed a written informed permission form, and the Medical Ethics Committee approved the study at Anhui Medical University’s Affiliated Stomatological Hospital. Each method was conducted as per appropriate regulations and guidelines.

HNSCC cell lines SCC9, CAL27, HN4, HN6, and human normal oral epithelial keratinocytes (HOK) were purchased from Ninth People’s Hospital Affiliated with Shanghai Jiaotong University School of Medicine in Shanghai (China). All cell lines were subjected to STR profiling and tested for *Mycoplasma* contamination every 3 months. These cell lines were saved in DMEM (BI, Israel) supplemented with 10% fetal bovine serum (BI, Israel), 1% penicillin, and streptomycin (NCM, Suzhou, China). Moreover, these cell lines were cultured at 37°C in a humidified incubator containing 5% CO_2_.

### Extraction of RNA and assessment of quality

Total RNA was extracted from frozen tumor tissues (C group) and matched non-cancerous tissues (N group) using Thermo Fisher Scientific’s TRIzol reagent (United States), according to the manufacturer’s instructions. Genomic DNA (gDNA) was extracted from tissues using PureLink Genomic DNA Mini Kit according to the manufacturer’s instructions (Thermo Fisher Scientific, K182001). A NanoDrop Spectrophotometer (Thermo Fisher Scientific, United States) was used to determine the concentration and purity of RNA. The OD value was measured after taking 1 μl RNA sample 50 times diluted, and the ratio of OD260/OD280 was greater than 1.8, indicating that the prepared RNA was pure and free from protein contamination. RNA samples were taken 1 μl, 1% agarose gel electrophoresis at 80 V × 20 min, EB staining for 10 min, observed and photographed with gel imaging system, the total RNA extraction was proved to be complete if the three bands were complete. RNA samples were stored at −80°C.

### Library preparation and sequencing

Libraries for the sequencing of were prepared from 2 μg of total RNA with the following modification. We removed ribosomal RNA using the Epicentre Ribo-Zero™ rRNA Removal Kit (Human/Mouse/Rat). Linear RNA was removed using RNase R (Epicentre, lnc). Magnetic beads with attached poly-T oligos were used to remove residual poly-A RNA. For fragmentation, divalent cations were used under increased temperature in an proprietary fragmentation buffer from Illumina. Using RNA as a template and primers of random oligonucleotides, synthesize cDNA first strand, then RNaseH was used to degrade the strand of RNA, and dNTP with dUTP instead of dTTP was used as the raw material to synthesize the cDNA second strand with in the DNA polymerase I system. Purify the cDNA, then perform double-end repair and introduce the “A” base at the 3′end and connect the sequencing adapter. At this time, USER enzyme (NEB, United States) was included in the system for the degradation of cDNA second strand of containing U. To select cDNA fragments of the preferred 0.4–0.5 Kbp length, purification of the library fragments was conducted using the AMPure XP system (Beckman Coulter, United States). Fragments of DNA having ligated adaptor molecules on the two ends were specifically enriched using the PCR Primer Cocktail from Illumina in a PCR reaction (15 cycle). After product purification (AMPure XP system) they were quantified through the Agilent high sensitivity DNA assay on Agilent’s Bioanalyzer 2100 system. This was followed by sequencing the library on Illumina’s NovaSeq 6000 platform (Shanghai Personal Biotechnology Cp. Ltd.).

### Differential expression analysis

To determine differentially expressed CricRNAs, DESeq (1.30.0) was used, and transcripts having |log2FoldChange| > 1 and *p*-value < 0.05 were considered differentially expressed CricRNAs. A heatmap package in R was used for the visual assessment of differentially expressed RNA. CSCD (http://gb.whu.edu.cn/CSCD/) is an online tool to study circRNAs specific to cancer and obtain probable structures of circRNA structures.

### Quantitative real-time polymerase reaction

By using the Prime Script RT Master Mix from Takara (Cat. #RR047A), the RNA (total) which were isolated from 20 samples were reversely transcribed. qRT-PCR was conducted in CFX96 Touch Real-Time PCR Detection System (Bio-Rad, Hercules, CA, United States) per the manufacturer’s protocol. For internal reference, GAPDH was used, and the reaction was conducted in two steps. The conditions for the reaction were: pre-denaturation at 95°C for 30 s; denaturation at 95°C for 5 s, and annealing/extension at 60°C for 30 s for 50 cycles. The threshold cycle (Ct) approach was used to estimate expression, and the 2^−ΔΔCt^ method calculated relative expression levels. Information about primers is shown in Table 1.

**TABLE 1:** A list of primers used in this study.

Gene	Primer	Sequence (5′ to 3′)
hsa_circ_0001394	Forward	TGG​AGA​ATG​ACG​ATG​ACC​CA
	Reverse	TTG​GTC​CAA​GGA​GAA​ACT​T
hsa_circ_0001821	Forward	CTCAGCTGGGCTTGAGGC
	Reverse	GTT​CCA​CCA​GCG​TTA​TTC​CC
hsa_circ_0004771	Forward	TCT​GAA​GAC​TCC​GGA​TGA​CA
	Reverse	TCA​CAA​TCC​AAA​CAC​TTC​CGT
hsa_circ_0005991	Forward	CCC​CGG​ATG​AAA​CAG​CTG​A
	Reverse	GCT​GTT​CCG​ATT​TGT​GAC​GT
hsa_circ_0060927	Forward	ATC​CAG​GCC​ACA​GAC​AAT​GA
	Reverse	CCA​GTC​TTC​CCC​TTC​CCT​GA
GAPDH	Forward	CAG​GAG​GCA​TTG​CTG​ATG​AT
	Reverse	GAAGGCTGGGGCTCATTT

### Competing endogenous RNA network construction

Based on the ceRNA theory, a circRNA-miRNA-mRNA regulatory network was prepared. The circBase database (http://www.circbase.org/cgi-bin/getseq.cgi) provided CircRNA information. Then, CircInteractome (https://circinteractome.nia.nih.gov/index.html) was used to determine DEcircRNA target miRNAs. These target miRNAs were crossed with DEmiRNAs in the OSCC patient dataset of the TGCA database using the Venn package, overlapping miRNAs were found, and finally, circRNA-miRNA pairs were established. We predicted the target mRNAs for intersecting miRNAs using Starbase (https://starbase.sysu.edu.cn) databases, miRDB (http://www.mirdb.org/mirdb/index.html), and TargetScan (http://www.targetscan.org/vert_72/), and only the genes that were found in all three databases at the same time were considered as likely mRNA targets. To obtain miRNA-mRNA pairs, the TCGA dataset was again used to intersect these target mRNAs with DEmRNAs. CirRNAs operate as miRNA molecular sponges, according to the ceRNA theory, and regulate mRNA expression. CircRNA expression is negatively connected with its target miRNA, while miRNA expression is adversely correlated with its target mRNA. A ceRNA regulatory network was prepared according to this principle using the above-identified circRNAs, miRNAs, and mRNAs. Finally, visualization of the ceRNA network was done using Cytoscape 3.8.2 software.

### Gene ontology and kyoto encyclopedia of genes and genomes enrichment analysis

On all mRNAs in the ceRNA network, KEGG (http://www.kegg.jp/) and GO (http://geneontology.org/) enrichment analyses were performed. Each of the genes was mapped to each term in the GO database, and for each term, the numbers of mRNAs were calculated using the hypergeometric distribution. GO terms with *p*-value corrected to ≤0.05 were deemed enriched significantly. KEGG automatic annotation server (KAAS) was applied for pathway annotation using the complete genome as the background. The hypergeometric distribution was applied to estimate the significant mRNAs enrichment pathway; a *p*-value ≤ 0.05 were deemed significantly enriched.

### Construction and validation of prognostic models

The association of DEmRNAs with overall survival (OS) and univariate Cox regression analysis were examined for the association between prognosis in patients with OSCC genes in the ceRNA network. Each DEmRNAs with *p*-value < 0.05 were finally chosen as a candidate gene for further analysis. To avoid overfitting, LASSO analysis was used, and the most appropriate prognostic DEmRNA was identified. After that, a multivariate Cox regression analysis was used to determine an optimal risk score. The risk score for patients with OSCC was estimated as follows: Risk score = $\sum_{i=1}^{n}X_{i}\times Y_{i}$ (where X_i_ is the risk factor and Y_i_ is the expression level of each gene, Supplementary Table S1). We exhibited ROC curves for the model at 1, 2, and 3 years and examined the values of the Acak Information Criterion (AIC) at each point of the one-year ROC curve to discover low- and high-risk Cutoff point scores. To confirm the validity of this cutoff, Kaplan-Meier survival analysis was used to determine the difference in survival between the two groups. R tools were used to visualize survival curves and risk scores for each patient.

A chi-square test was used to verify the practicability of the generative model for clinical application and assess the association between clinicopathological features and risk scores. Band plots were drawn for visualization. The Wilcoxon signed-rank test was also used to see any differences in risk ratings across groups depending on clinical features, with the results displayed in box plots.

### Assessment of tumor-infiltrating immune cells

To examine if there was a link between immune cells and risk scores in the tumor microenvironment, we employed well-established approaches such as TIMER, XCELL, EPIC, QUANTISEQ, CIBERSORT, MCPCOUNTER, and CIBERSORT-ABS to analyze TICs in samples. The relationship between risk scores and TICs was estimated by Spearman correlation analysis, and the correlation coefficient is shown in the lollipop plot (significance threshold *p* < 0.05). The R ggplot two package uses this operation.

### Investigation of the expression of ICI-Related molecules

The “limma,” “reshape2,” “ggplot2,” and “ggpubr” packages in R were used to examine the expression of immune checkpoint genes in high-risk and low-risk patients, and immunotherapy score data was received from TCIA. To further evaluate the prognostic function of our risk model, sensitivity to immunotherapy was assessed for patients in high-risk and low-risk groups.

### Chemotherapeutic drug sensitivity analysis

To assess the value of features in predicting treatment effect in OSCC, we calculated IC50s for standard chemotherapeutics and molecularly targeted drugs per sample applying pRRophetic. Guidelines recommend using antitumor drugs such as gefitinib, doxorubicin, gemcitabine, and rapamycin to treat OSCC. The Wilcoxon signed-rank test compared IC50 differences between high-risk and low-risk groups, and we used the “ggplot2” R package to show the results as box plots.

### Cell transfection

We employed a negative control (NC) oligonucleotide and short interfering RNA (siRNA) to target hsa_circ_0005991. For cell culture, CAL27 cells were seeded in 6-well plates. Then, using Liposome 2000 (Invitrogen, United States) by the manufacturer’s instructions, siRNAs or controls (General, Anhui, China) were added to the cells at a final concentration of 50 nM.

### Cell counting kit-8 proliferation assay

In each well of a 96-well plate, a total of 3,000 cells were implanted. 10 ul of CCK-8 reagent was added to the culture medium at 0, 24, 48, and 72 h after transfection. Then, the cells were incubated at 37°C for 1 h, and the absorbance was measured at 450 nm with a microplate reader.

### Wound healing assay

Eighty percent fusion was achieved after transfected CAL27 cells were injected onto a 12-well plate. With the tip of a 10 ul pipette, single-cell layers were scraped. After three PBS washes to remove cell debris, fresh media containing serum was added. Three high magnification fields were taken at 0 h and 24 h after scratching to obtain typical images of cell migration. Using ImageJ, the scratch width was estimated.

### Transwell migration and invasion assays

In the migration assay, transfected CAL27 cells were inoculated in the upper chamber of the Transwell system (BD Biosciences, San Jose, CA, United States), and the lower chamber was filled with 500 μl of medium containing 10% FBS. 24 h later, the cells remaining on the surface of the filter membrane were gently wiped off with a cotton swab, and the cells passing through the membrane were fixed with methanol and then stained with crystal violet solution. Under an inverted microscope, three randomly selected fields of view (including the center and periphery of the membrane) were used to count the number of cells. Matrigel (BD Biosciences, San Jose, CA, United States) was used to coat the filters of the Transwell system in the invasion assay, and the other steps were the same as for the migration assay.

### Statistical assessments

Each experiment was repeated thrice, independently. Data are presented as the mean ± SD (standard deviation). For data analysis, the two-tailed Student’s *t*-test was applied. *p* < 0.05 was deemed a statistically significant difference.

## Results

### CircRNA profiles differentially expressed in OSCC

**FIGURE 1:**
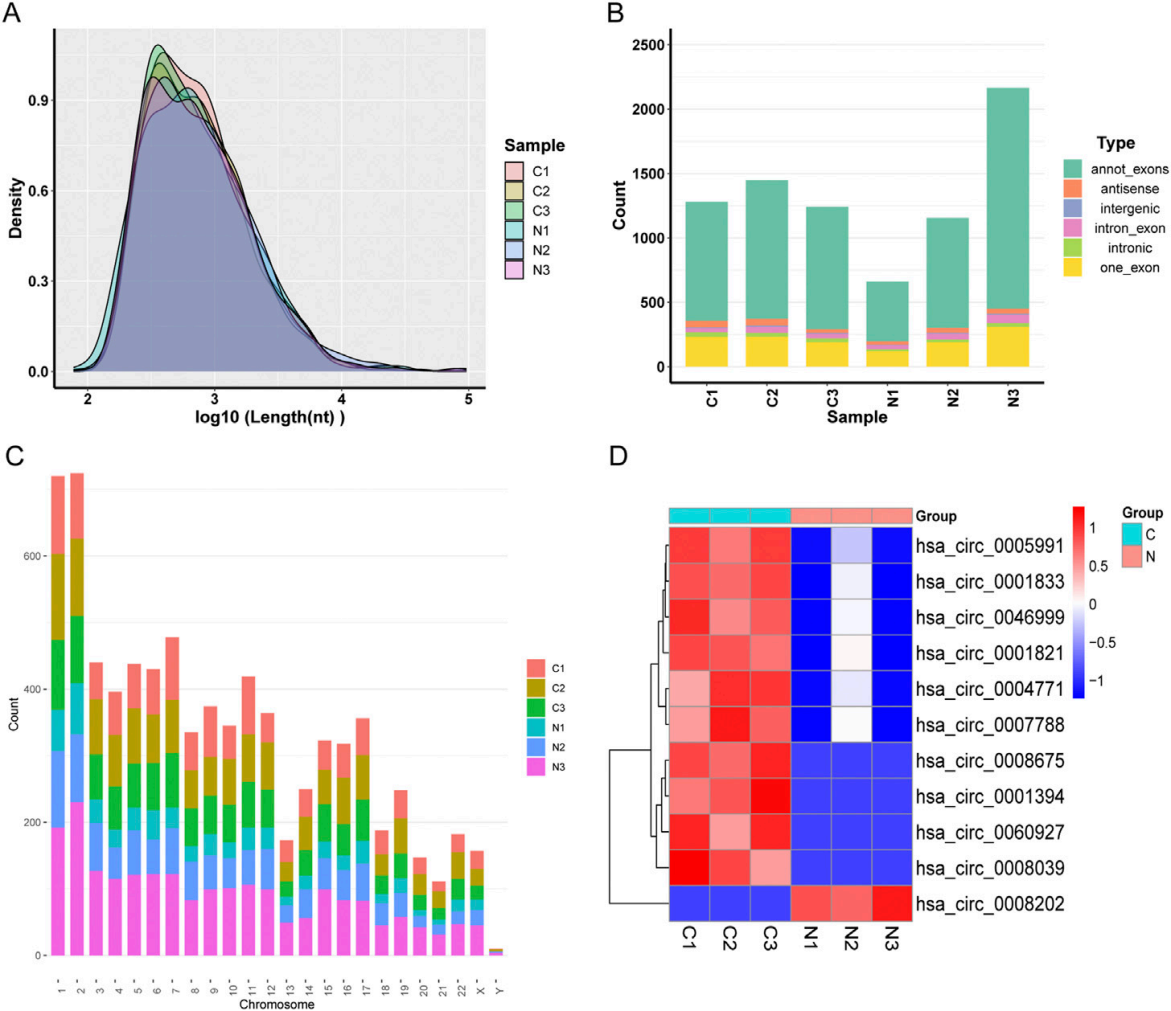
**(A)** Distribution of the lengths of the detected circRNAs. The length of the identified circRNAs is shown on the *x*-axis, and the abundance of circRNAs sorted by length is shown on the *y*-axis. **(B)** The circRNA category is depicted as a bar diagram. **(C)** In human chromosomes, the distribution of differently expressed circRNAs. **(D)** Analysis of all target circRNAs using a heat map and hierarchical clustering. High relative expression is shown by the red strip, whereas low relative expression is represented by the blue strip.

We discovered many circRNA in the neighboring normal tissue and three pairs of OSCC samples. These samples revealed a total number of targets of 3,738 circRNAs, including 2,421 known circRNAs and 1,317 new circRNAs. The lengths of candidate circRNAs were mostly <2,000 nucleotides (nt) (Figure 1A). The majority of circRNA is transcribed from exons that code for proteins. Some are intragenic and antisense and come from introns (Figure 1B). Candidate circRNAs are found on all chromosomes but primarily concentrate on the first and second (Figure 1C). CircRNAs that were differentially expressed with a statistical significance among the two groups were identified with |log2FoldChange| > 1 and *p* < 0.05. 11 circRNAs were significantly differentially expressed and visualized by a clustered heatmap (Figure 1D). In these samples, ten molecules of circRNA were up-regulated, and one was down-regulated (Table 2). We visualized the structure of circRNAs according to the CSCD database, and the results showed that all 11 circRNAs have miRNA response elements (MREs) (Supplementary Figure S1).

**TABLE 2:** 11 differently expressed circRNAs in oscc.

CircRNA ID	Chr	Locus	Strand	Length	Type	Source gene	Regulation
hsa_circ_0004771	21	16386664–16415895	−	203	Exons	NRIP1	Up
hsa_circ_0060927	20	52773707–52788209	−	1,106	Exons	CYP24A1	Up
hsa_circ_0005991	4	41015599–41016415	−	816	One_exon	APBB2	Up
hsa_circ_0008039	7	716865–751164	−	462	Exons	PRKAR1B	Up
hsa_circ_0001394	4	6925099–6925838	+	739	One_exon	TBC1D14	Up
hsa_circ_0008675	20	47886478–47888287	−	1,809	One_exon	ZNFX1	Up
hsa_circ_0001821	8	128902834–128903244	+	410	Exons	TCONS_00015354	Up
hsa_circ_0007788	16	4516153–4519466	−	489	Exons	NMRAL1	Up
hsa_circ_0046999	18	12999419–13042333	+	2,071	Exons	CEP192	Up
hsa_circ_0001833	8	145245686–145255444	+	579	Exons	HEATR7A	Up
hsa_circ_0008202	1	48821341–48825442	−	285	Exons	SPATA6	Down

### Differentially expressed circRNAs validation by qRT-PCR

**FIGURE 2:**
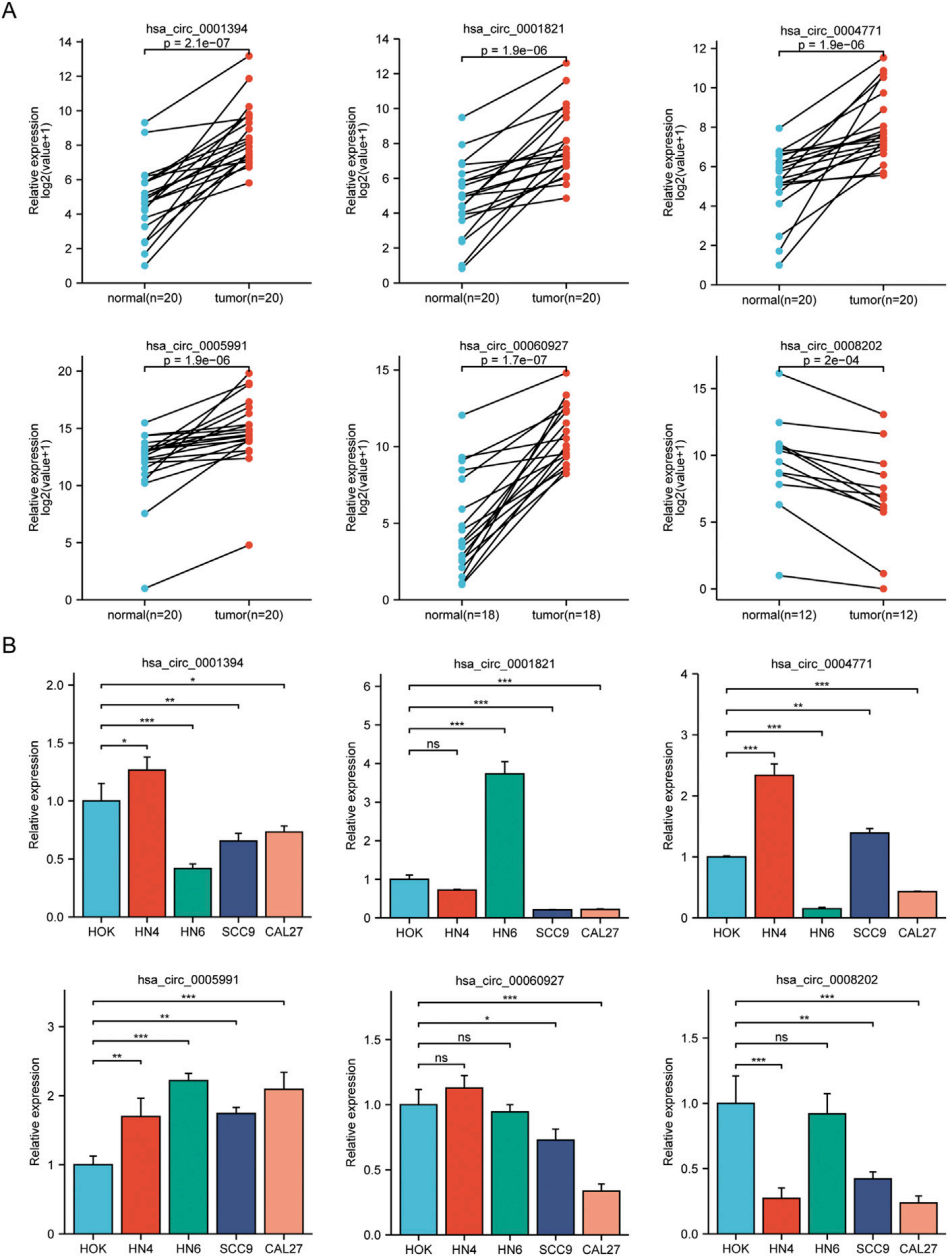
Analysis of circRNAs in cell lines and OSCC tissues. **(A)** Results of qRT-PCR in clinical specimens for six circRNAs. Five circRNA molecules were significantly upregulated, and one circRNA molecule was significantly downregulated in tumor tissues compared to normal tissues. **(B)** The expression of six circRNAs in OSCC cell lines (HN4, HN6, SCC9, CAL27) compared with HOK. *p < 0.05, **p < 0.01, ***p < 0.001. NS, no significance.

We conducted qRT-PCR in 20 sample pairs that included the analysis of tissues for high-throughput sequencing. The results showed that five circRNA molecules were significantly upregulated, and one circRNA molecule was significantly downregulated in tumor tissues compared to normal tissues (Figure 2A). Figure 2B shows the validation results of the above six circRNA molecules in OSCC cell lines.

### Prediction of the circRNA–miRNA–mRNA interaction network

**FIGURE 3:**
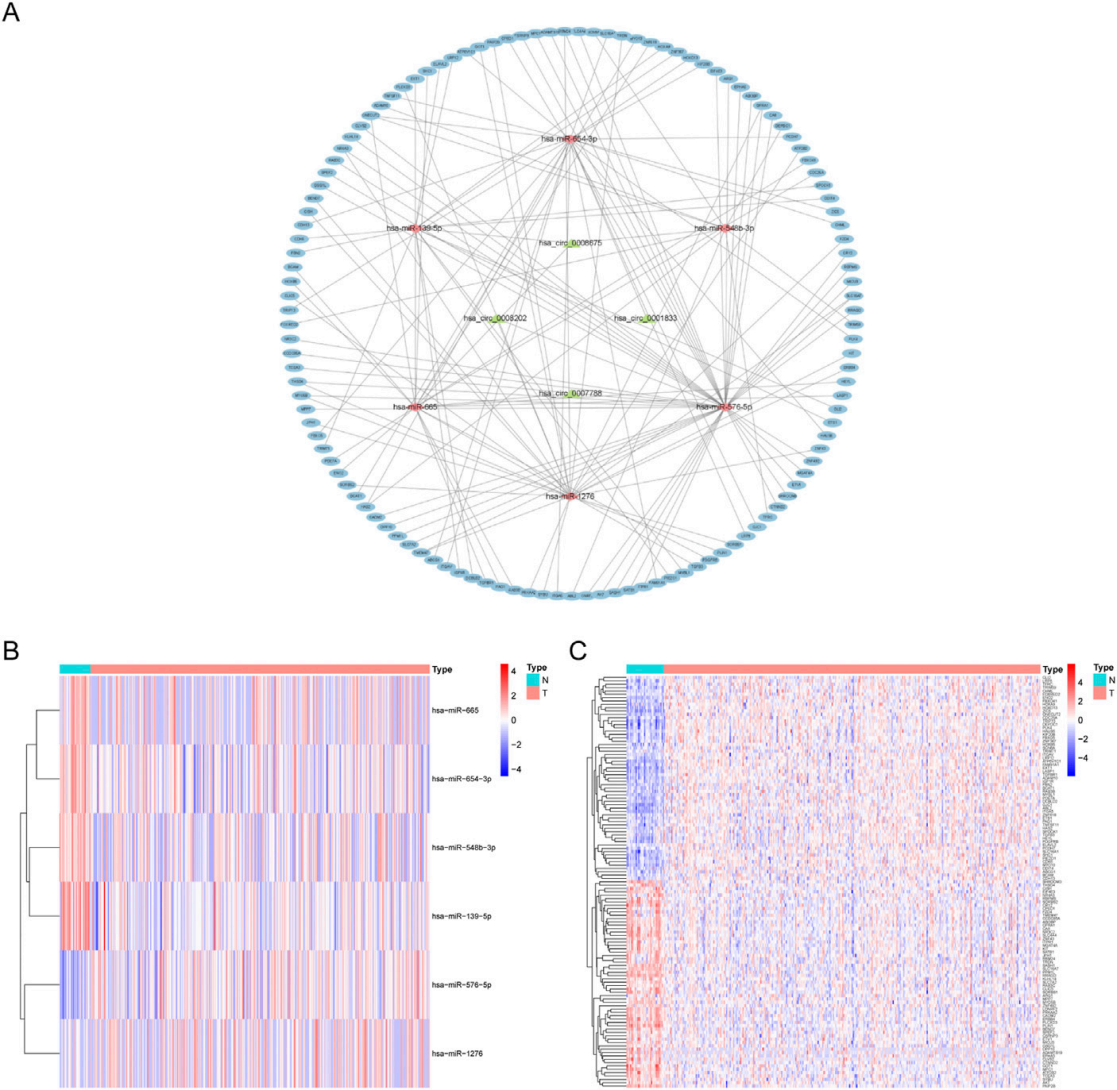
CeRNA network construction and node differential expression. **(A)** The ellipse represents 123 mRNAs, the diamond six microRNAs, the triangle four circular RNAs. **(B,C)** The differential expression of miRNAs and mRNAs in the ceRNA network in OSCC tissues and adjacent tissues in the TCGA database. N, adjacent non-tumor tissues; T, tumor tissues.

To learn more about the functions of mRNAs and circRNAs in OSCC incidence, we built a ceRNA network. The ceRNA network will only include genes that match the following criteria: 1) All genes should be expressed differentially; 2) circRNAs and mRNAs should have an association with miRNAs through binding at the same time; and 3) RNAs (circRNAs, mRNAs) and miRNAs must be negatively regulated. We finally identified four circRNAs (3 upregulated and one downregulated), six overlapping miRNAs (2 upregulated and four downregulated), and 123 overlapping mRNAs (61 upregulated and 62 downregulated) to construct a ceRNA network (Figure 3A). Multiple circRNAs can operate as ceRNAs, capturing downstream miRNAs and influencing phenotypes through mRNA regulation. In addition, we performed a differential analysis of the nodes in the network and drew a heat map based on the expression data in the TCGA database. The results showed significant differences between the node molecules in cancer and paracancerous tissues (Figures 3B,C). Then, Kaplan-Meier survival analysis was used to validate further the association between genes involved in the ceRNA network and OSCC prognosis. The results showed that a total of 65 genes were significantly associated with overall survival in OSCC (*p* < 0.05, Supplementary Table S2).

### Analysis of kyoto encyclopedia of genes and genomes pathway and annotation of gene ontology function

**FIGURE 4:**
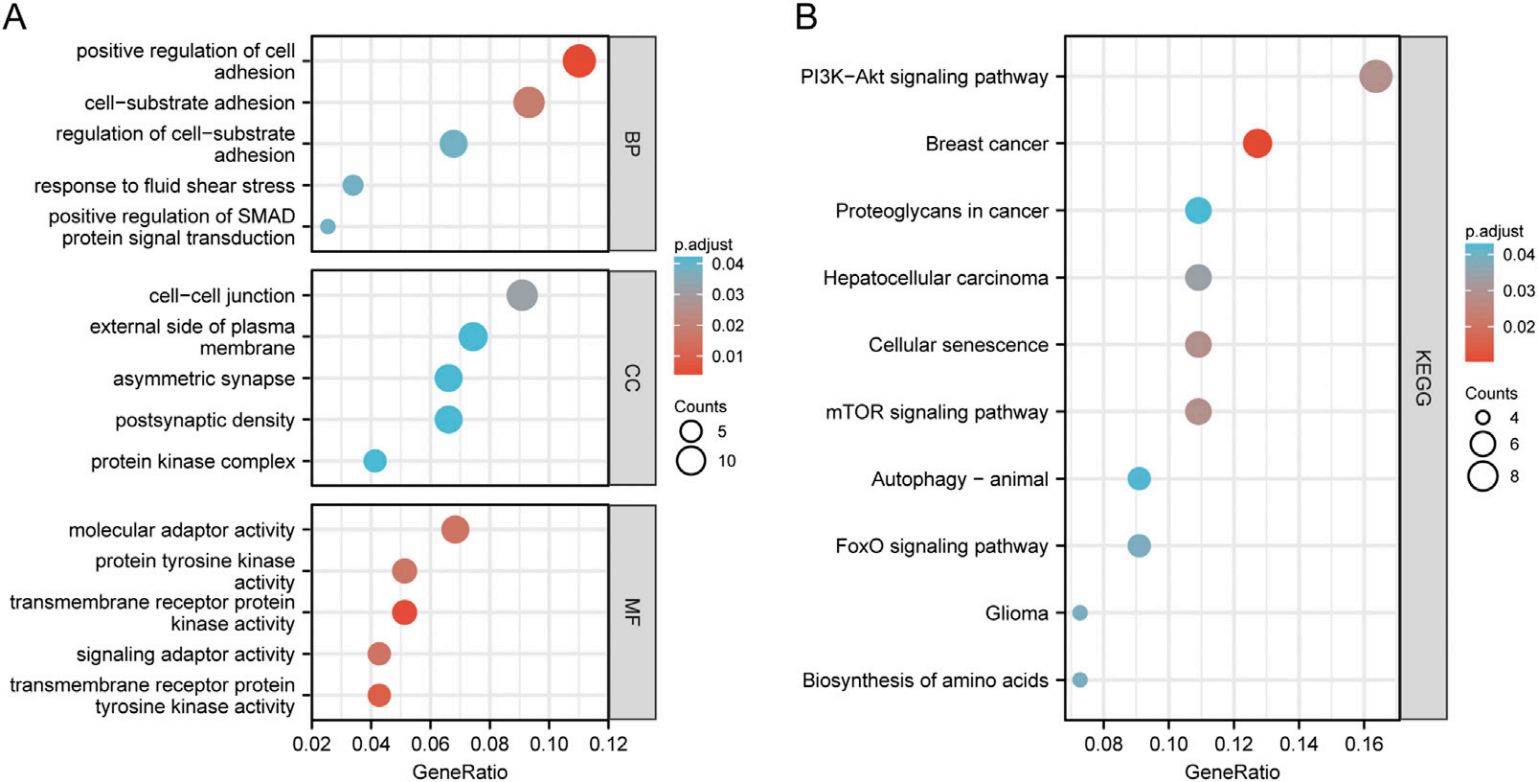
Enrichment analysis of function. Bubble plot of target gene GO enrichment analysis: **(A)** BP, CC, and MF in the ceRNA network; **(B)** The bubble chart of the signal pathway focused by the KEGG enrichment analysis of genes in the ceRNA network.

We conducted KEGG and GO functional enrichment analysis on mRNAs (*n* = 123) in the ceRNA network (Supplementary Table S3). The top five enriched CC (Cellular Component), BP (Biological Process), MF (Molecular Function) terms, and KEGG pathways are presented in Figures 4A,B. They are enriched mainly in positive regulation of cell adhesion, positive regulation of SMAD protein signal transduction (BPs), the cell-cell junction (CCs), and transmembrane receptor protein kinase activity (MFs). Analysis of the KEGG pathway shows enrichment in the PI3K-Akt signaling pathway, mTOR signaling pathway, cellular senescence, and proteoglycans in cancer. These findings suggest that the differentially expressed genes are linked to tumor signaling pathways and OSCC progression.

### Construction and evaluation of risk assessment model

We found that seven DEmRNAs substantially impacted patients’ overall survival by applying univariate Cox regression (*p* < 0.05, Supplementary Table S4), suggesting that mRNAs in the ceRNA prognostic subnet might affect the survival and prognosis of OSCC patients. Following that, LASSO Cox regression analyses were utilized to establish a risk model for the above seven genes, and the LASSO regression analysis results kept the above seven genes (Supplementary Figures S2A,B). Finally, a multivariate Cox regression analysis was performed using the stepwise regression approach, and seven DEmRNAs were screened out to establish an OSCC prognostic model (Supplementary Figures S2C,D).

**FIGURE 5:**
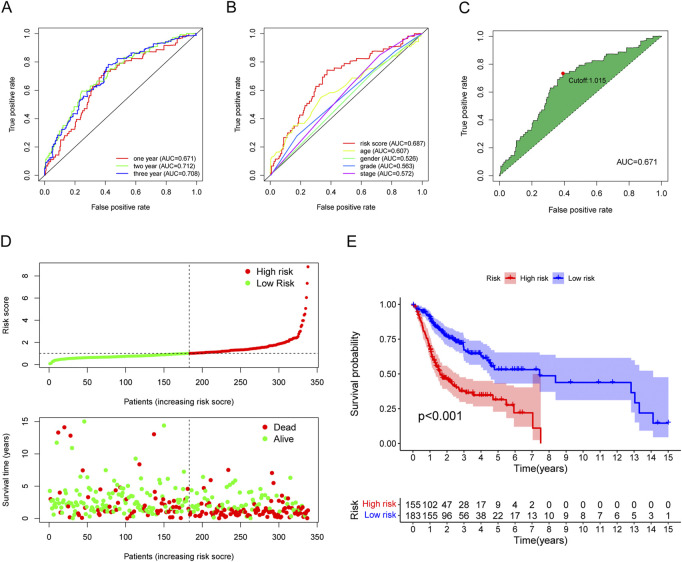
Evaluation of the risk assessment model for prognosis prediction. **(A)** The AUCs of the 1-, 2-, and 3-year ROC curves. **(B)** The 1-year ROC curve of the risk model and other clinicopathological characteristics. **(C)** The cut-off risk score identified by Youden Index. **(D)** The distribution of risk score and survival time and status. **(E)** The overall survival of the low-risk groups was higher based on K-M analysis.

To verify the model’s accuracy, ROC curves (1-, 2-, and 3-year) were drawn, and could successfully prognosticate OSCC patients because all values of areas under curve (AUC) were more than 0.65 (Figure 5A). Next, with the help of multi-metric ROC curves, we plotted the ROC curves of the risk model together with the ROC curves of clinical characteristics such as age, sex, grade, and stage in the same graph for comparison. The results showed that the AUC values of the risk model were significantly better than the clinical parameters, indicating the model’s high performance (Figure 5B). We also used the AIC value for the identification of the maximum inflection point as the cutoff point for the ROC curve for 1-year (Figure 5C).

### Model for clinical evaluation through risk assessment

**FIGURE 6:**
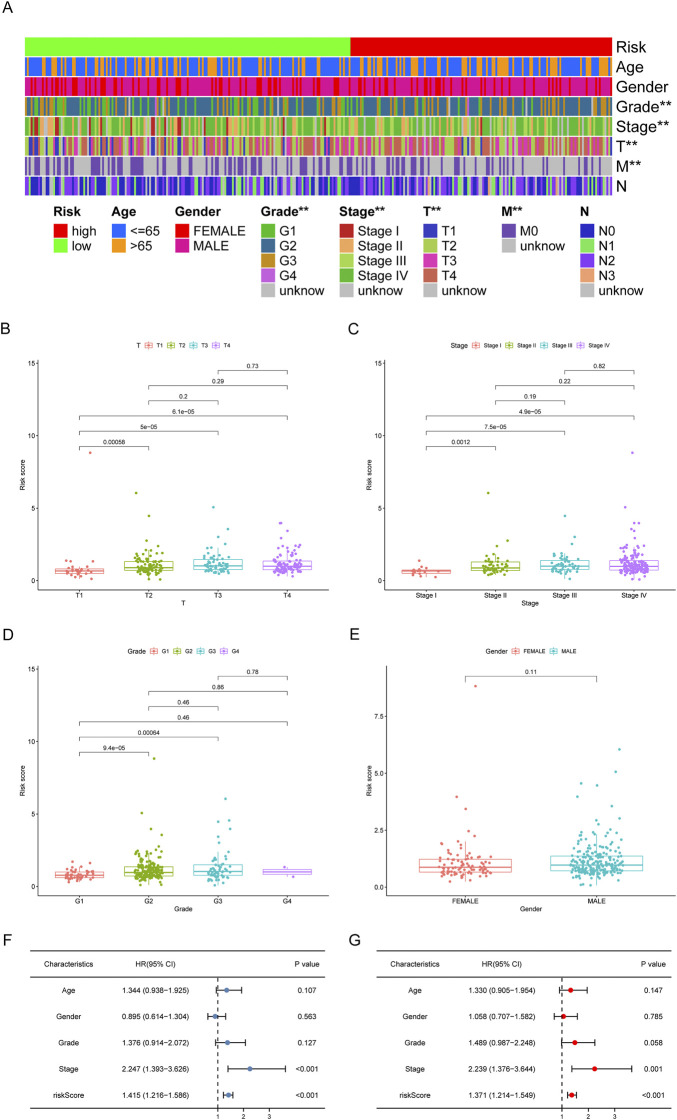
Clinical evaluation of the risk assessment model. **(A)** The strip chart and the scatter diagram demonstrated that T stage **(B)**, clinical stage **(C)**, tumor grade **(D)**, and gender **(E)** were significantly correlated with the risk score. *p < 0.05, **p < 0.01. **(F,G)** The risk model could be used as an independent prognostic predictor by the univariate and multivariate Cox regression analysis.

Based on the earlier determined cut-off points, 155 cases were classified as a high-risk group, and 183 were classified as a low-risk group. The risk score distribution for each OSCC case is presented in Figure 5D; these data indicate better clinical outcomes for low-risk group patients than those in the high-risk group. Patients with OSCC in the high-risk group exhibited a significant decrease in survival than low-risk OSCC patients, according to a Kaplan-Meier analysis and associated survival curve (*p* < 0.001; Figure 5E). In addition, the analysis showed that nearly all patients with high-risk scores lived under 8 years, while in the low-risk group, about 50% were still alive. We used chi-square tests to assess whether the risk model is associated with the clinicopathological traits of OSCC patients. Bar graphs show overall results, with tumor grade and the stage being exceptionally closely related to risk (Figure 6A). The proportion of each clinicopathological feature in the high- or low-risk group is shown in Supplementary Figure S3. In addition, we analyzed the differences in risk scores between groups stratified by different clinicopathological factors. As shown in Figures 6B–D, statistically significant high-risk scores were more common in patients with higher tumor grades and more advanced clinical stages. However, there were no differences in risk scores between patients by gender (Figure 6E). Finally, we performed univariate and multivariate cox analyses and found that the risk model could be used as an independent prognostic predictor (Figures 6F,G). In conclusion, a risk assessment model based on DEmRNAs can be used as a reliable predictor of survival outcomes and tumor aggressiveness in OSCC.

### Correlation between tumor-infiltrating immune cells or immunosuppressive molecules and the risk model

**FIGURE 7:**
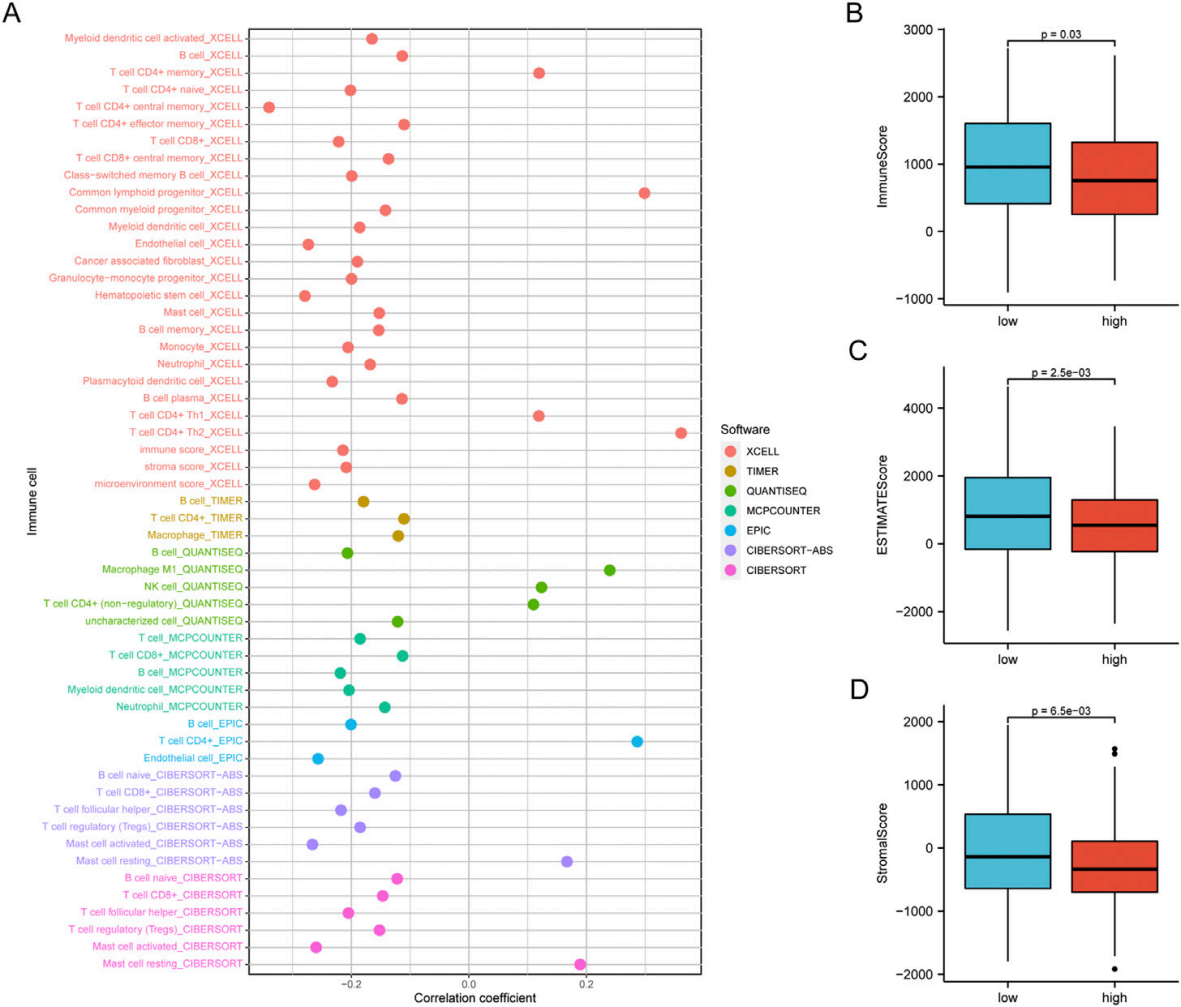
The Risk assessment model was used to estimate tumor-infiltrating cells. **(A)** According to Spearman correlation analysis, patients in the high-risk group were more positively associated with tumor-infiltrating immune cells like ommon lymphoid progenitor, macrophages, NK cells, while they were negatively associated with CD4^+^ T cells, B cells, CD8^+^ T cells, mast cells, and neutrophils. **(B)** The low-risk group has higher TME scores.

**FIGURE 8:**
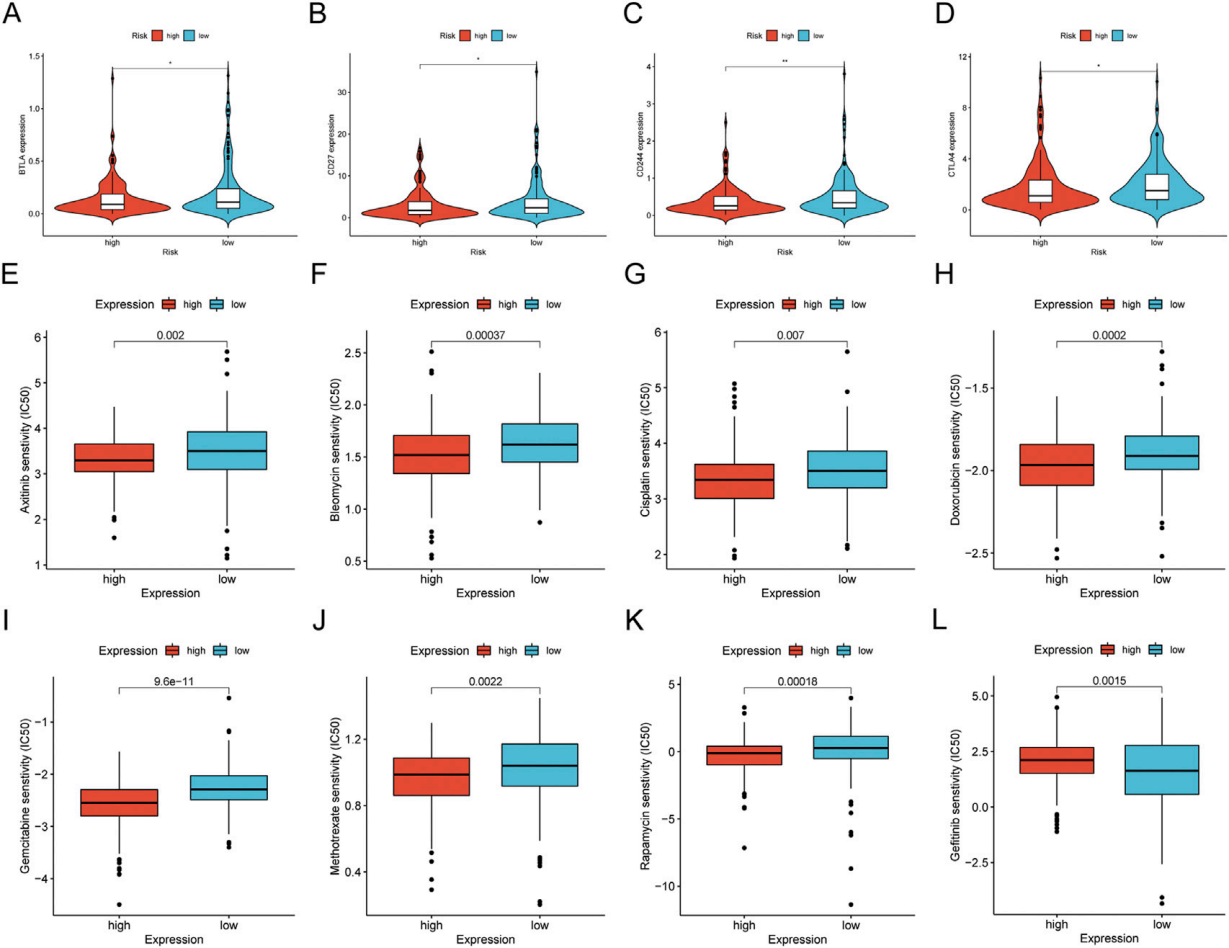
Differences of checkpoint-related gene expression and chemotherapeutic sensitivity in high- and low-risk groups. **(A–D)** The high-risk group in the model was negatively associated with BTLA, CD27, CD244, and CTLA4. The high-risk group in the model was related to lower IC50 of chemotherapeutics of axitinib **(E)**, bleomyci **(F)**, cisplatin **(G)**, doxorubicin **(H)**, gemcitabine **(I)**, methotrexate **(J)**, rapamycin **(K)**, and the low-risk group was related to lower IC50 of chemotherapeutics of gefitinib **(L)**.

We concentrated on immune cell infiltration and looked at the model’s connection with the tumor immunological microenvironment. According to Spearman correlation analysis, a high-risk group in the model was correlated positively with TICs, including common lymphoid progenitors, macrophages, and NK cells. In contrast, CD4^+^ T cells, CD8^+^ T cells, neutrophils, mast cells, and B cells had a negative correlation (Figure 7A). In addition, compared to the low expression group, the low-risk group was significantly higher in the stromal score, immune score, and ESTIMATE score (Figures 7B–D). We further investigated whether the model was associated with ICI and found that the high-risk group in the model was negatively associated with various immune checkpoint molecules, such as BTLA, CD27, CD244, and CTLA4 (Figures 8A–D).

### Analysis of the correlation between chemosensitivity and the risk mode

We investigated common medication sensitivities (expressed as IC50s) in individuals with low and high-risk ratings to see if the efficacy of several regularly used chemotherapeutic medicines is associated with risk. The patients with high-risk had a lower IC_(50) for axitinib (*p* = 0.002; Figure 8E), bleomycin (*p* = 0.00037; Figure 8F), cisplatin (*p* = 0.007; Figure 8G), doxorubicin (*p* = 0.0002; Figure 8H), gemcitabine (*p* < 0.0001; Figure 8I), methotrexate (*p* = 0.0022; Figure 8J), rapamycin (*p* = 0.00018; Figure 8K), higher IC_(50) for gefitinib (*p* = 0.0015; Figure 8L). Therefore, this model can potentially predict sensitivity to chemotherapeutic drugs.

### Hsa_circ_0005991 promotes HNSCC cells proliferation, migration, and invasion *in vitro*

**FIGURE 9:**
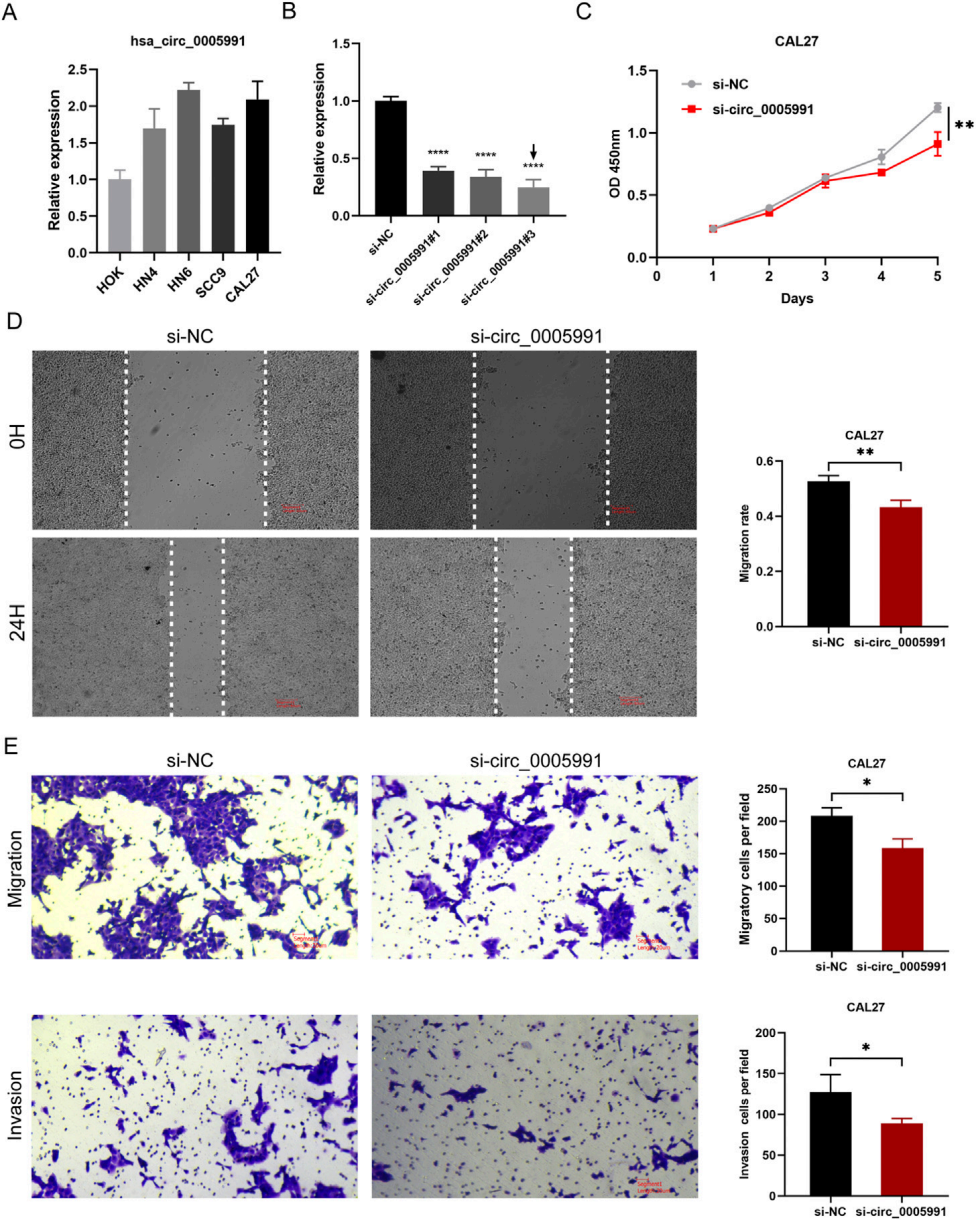
Hsa_circ_0005991 promotes the proliferation, migration and invasion of HNSCC cells *in vitro*. **(A)** The qRT-PCR results revealed that HOK cells expressed hsa_circ_0005991 at a lower level than five HNSCC cells. **(B)** qRT-PCR analysis of hsa_circ_0005991 expression in CAL27 cells treated with siRNAs. **(C)** The proliferation ability of CAL27 cells transfected with NC or si-hsa_circ_0005991 was examined by CCK-8 assay. **(D,E)** The migration and invasion ability of the cells were detected by wound healing and transwell assays. Data were showed as mean ± SD,*p < 0.05, **p < 0.01, ***p < 0.001, ****p < 0.0001.

In HOK and five HNSCC cell lines, the expression of hsa_circ_0005991 was identified by qRT-PCR, and the results revealed that four HNSCC cells expressed hsa_circ_0005991 at a higher level than HOK cells (Figure 9A). Ultimately, the CAL27 cell lines were selected by us to represent the knockdown cells of hsa_circ_0005991. In CAL27 cells treated with siRNA fragments, according to qRT-PCR data, hsa_circ_0005991 expression was dramatically down-regulated. The si-hsa_circ_0005991-3 was chosen for further investigation among the three siRNAs because it showed the best silencing effectiveness in CAL27 cells (Figure 9B). The downregulation of hsa_circ_0005991 inhibited the proliferative activity of HNSCC cells according to the CCK-8 assay (Figure 9C). Then, the influences of hsa_circ_0005991 on HNSCC cell invasion and migration were examined by wound healing and transwell assays. The results showed that downregulation of the hsa_circ_0005991 gene inhibited CAL27 cells invasion and migration ability (Figures 9D,E). These experiments proved that in HNSCC cells, hsa_circ_0005991 could promote proliferation, migration, and invasion.

## Discussion

Researchers in different countries have made significant progress in OSCC research in recent years, especially in developing molecularly targeted drugs and antibody-drug conjugates, which have greatly benefited OSCC patients (Nagaya et al., 2017; Ketabat et al., 2019; Zhang et al., 2020). Current treatments for OSCC include surgery, chemotherapy, molecularly targeted therapy, and radiation therapy. Although there are various treatment methods, most OSCC patients have entered the advanced stage, and the overall treatment effect is not ideal due to factors such as drug resistance, distant metastasis, and poor typing. The pathogenesis and progression of cancer are closely related to genetic mutations and genetic diseases. Studies have shown that dysregulation of circular RNA expression plays a vital role in the pathogenesis and progression of many tumors. CircRNAs are suggested to express in a tissue-specific and developmental stage-specific manner, which makes them potential biomarkers of specific diseases (Wang et al., 2021). CircRNA-associated ceRNA regulatory networks play critical roles in the pathogenesis and progression of OSCC, bladder cancer, colorectal cancer, and other cancers (Song and Fu, 2019; Wu et al., 2019; Liu et al., 2020). Importantly, mRNAs, miRNAs, and circRNAs in ceRNA networks can serve as potential targets for OSCC therapy and molecular targets for assessing prognosis. However, little is known in the OSCC field about the combined analysis of circRNA-based ceRNA networks and the immune microenvironment. Therefore, we constructed a circRNA-miRNA-mRNA network and analyzed target genes and prognostic, TIC, and immune checkpoints to identify novel molecular mechanisms and prognostic biomarkers of OSCC occurrence. We also aimed to explore new targets that could mediate specific tumor immunotherapy.

We found abundant expression of circRNAs in three pairs of samples, including 2421 existing circRNA molecules and 1,317 new circRNA molecules. Most of the circRNAs were derived from exons. Exonic circRNAs are generated by a process called reverse slicing, which is a disorderly arrangement of exons (Zhang et al., 2013). This study identified ten up-regulated and one down-regulated circRNA molecule, and six selected circRNAs were consistent with RNA-seq data after validation using qRT-PCR.

Hsa_circ_0004771, hsa_circ_0005991, and hsa_circ_0060927 are spliced by nuclear receptor interacting protein 1 (NRIP1), amyloid beta precursor protein binding family B member 2 (APBB2), and cytochrome P450 family 24 subfamily A member 1 (CYP24A1), respectively, which play essential roles in tumor proliferation, migration, and apoptosis. NRIP1, a coregulator of several nuclear receptors and transcription factors, can act as a co-activator or corepressor and is upregulated in a variety of tumor types, including gastric cancer (Liang and Li, 2020), gastric adenocarcinoma (Fang and Lu., 2020), esophageal squamous cell carcinoma (Ni et al., 2018), and breast cancer (Binato et al., 2021). APBB2 is an articulated protein characterized for its function in amyloid precursor protein processing (Tanahashi and Tabira, 2002). APBB2 plays a dual regulatory role in bladder cancer, mediating the cell cycle through the CDK6 and MET pathways (Li et al., 2019). CYP24A1 is a mitochondrial enzyme responsible for the inactivation of vitamin D. Fazeli et al. (2020) demonstrated for the first time that hsa_circ_0060927 is ectopically expressed in uterine smooth muscle tumors compared to adjacent tissues.

In addition, a ceRNA network consisting of four circRNAs, six miRNAs, and 123 mRNAs was constructed based on DEcircRNA. The biological functions of DEmRNA were analyzed by GO functional annotation and KEGG pathway enrichment. The results of GO analysis revealed that cell adhesion participates in the functions of these DEmRNAs, and the role of cell adhesion in the progression of OSCC has been extensively studied (de Campos et al., 2017; Kita et al., 2017; Aseervatham and Ogbureke., 2020). KEGG analysis showed that DEmRNAs are widely involved in the PI3K-AKT signaling pathway, mTOR signaling pathway, and Cellular senescence. These pathways play a crucial role in the development of OSCC. The PI3K-AKT-mTOR signaling pathway is associated with several cellular functions, controlling protein synthesis, cell growth, apoptosis, proliferation, and angiogenesis, and may regulate the progression of OSCC (Martins et al., 2016). Wu et al. (2022) found that HPRT1 could improve cisplatin (CDDP) resistance in OSCC patients by promoting the MMP1/PI3K/Akt axis. Liu et al. (2022) showed that LHPP promotes apoptosis in OSCC by decreasing the transcriptional activity of p-PI3K and p-Akt.

A prognostic risk model with seven genes was developed by integrating LASSO regression and Cox regression analysis. Kaplan-Meier curve analysis showed that our prognostic model could accurately distinguish between high- and low-risk populations. Furthermore, the AUC of the ROC plots for 1-year, 2-year, and 3-year overall survival were 0.671, 0.712, and 0.708, respectively. The results confirmed the excellent predictive value of our risk profile. Of these seven genes, six are potential risk genes, and one is a potential protective gene. Six genes are involved in the development and progression of various cancers. LIM and SH3 protein 1 (LASP1) is a member of the LIM family of proteins, initially identified from a cDNA library of human breast cancer tissue (Tomasetto et al., 1995). LASP1 expression was reported to be increased in OSCC patients and significantly correlated with primary tumor size. Further studies have shown that LASP1 promotes OSCC cell proliferation by accelerating cell cycle progression (Shimizu et al., 2013). DEP domain containing 1 (DEPDC1) is a highly conserved protein in many species, from *Cryptobacterium* hidradenum to mammals (Kanehira et al., 2007). Guo et al. (2020) showed that DEPDC1 exerts its oncogenic activity by suppressing CYP27B1 protein expression, while NNK promotes DEPDC1 upregulation by stimulating DNMT1 expression in OSCC. Similarly, DNA damage-inducible transcript 4 (DDIT4) and glutamic-oxaloacetic transaminase 1 (GOT1) as oncogenes are associated with poor prognosis in OSCC (Busso-Lopes et al., 2021; Han et al., 2021). Basal cell adhesion molecule (BCAM), also known as Lutheran, is widely expressed in various tissues and involves many biological processes, such as cell adhesion, migration, and invasion (De Grandis et al., 2013; Bartolini et al., 2016). Emerging research suggests that BCAM plays a vital role in tumor progression, including skin tumors, hepatocellular carcinoma, colorectal cancer, and bladder cancer (Bartolini et al., 2016; Chang et al., 2017; Jin et al., 2020). Choi et al. (2020) identified a novel tumor suppressor, KLHL14, a subunit of ubiquitin ligase, associated with the endoplasmic reticulum-associated protein degradation (ERAD) machinery and is recurrently mutated in mature B cell malignancies. However, the roles of KLHL14, BCAM, and HAUS augmin like complex subunit 6 (HAUS6) in OSCC, are unknown.

Since a single gene may predict OS instability, a signature integrating the efficacy of seven immune-related genes would show strong predictive power. To establish a relatively accurate prognostic model in OSCC patients, this study proposes a novel prognostic line chart, including immune-related risk scores and clinicopathological features, which showed good predictive power. To explore the relationship between risk scores and tumor-infiltrating immune cells, we used seven commonly accepted methods to estimate immune infiltrating cells, including XCELL (Aran et al., 2017; Aran, 2020), TIMER (Li et al., 2017; Li et al., 2020), QUANTISEQ (Finotello et al., 2019; Plattner et al., 2020), MCPCOUNTER (Dienstmann et al., 2019), EPIC (Racle et al., 2017), CIBERSORT ABS (Tamminga et al., 2020), and CIBERSORT (Chen et al., 2018; Zhang et al., 2020). Analyzing these results, we found that the high-risk population in the model was significantly and negatively associated with immune infiltration of CD8^+^ T cells, neutrophils, mast cells, and B cells. The role of B cells has been controversial: some studies have shown that B cell infiltration in tumors is associated with poor prognosis, while others have shown the opposite (Tsou et al., 2016; Sarvaria et al., 2017). CD8^+^ T cells are the major lymphocyte subpopulation that kills cancer cells with major histocompatibility class I molecules (Shen and Ren., 2018). The frequency of CD8^+^ T cells was positively correlated with survival in patients with lung cancer, melanoma, and breast cancer (Reiser and Bamerjee., 2016; van der Leun et al., 2020). In addition, infiltration of CD8^+^ T cells in TME was associated with improved response in patients with ICIS-treated cancers. Wong et al. (2019) found that melanoma patients with high CD8^+^ T cell counts survived longer on anti-PD-1 therapy. Theoretically, neutrophils may be a potent antitumor effector cell because neutrophil granules contain various antimicrobial and cytotoxic compounds that can destroy malignant cells (Ocana et al., 2017). However, many studies have shown that tumor-associated neutrophils may promote tumor progression. N2 Polarized neutrophils morphologically resemble granulocytes or polymorphonuclear myeloid-derived suppressor cells and thus may exert tumor suppressive effects (Rakic et al., 2018). Although the signature is associated with checkpoint-associated biomarkers such as BTLA, CD27, CD244, and CTLA4, we believe that the specific mechanisms and biomarkers are yet to be confirmed and validated due to differences in different immune cells and immune-associated phenotypes. To further evaluate the value of risk models for clinical application in the treatment of OSCC, we calculated the IC50 of several commonly used antitumor drugs and compared the differences in drug sensitivity between patients in the high-risk and low-risk subgroups. We found that high-risk OSCC patients had higher sensitivity (lower IC50) to antitumor drugs such as cisplatin, gemcitabine, and rapamycin. The relationship between the risk model and drug sensitivity can be used to guide the selection and administration of clinical antitumor drugs and needs to be further investigated. Because our conclusions are based on computational predictions, molecular biology tests are required to ascertain the role of CircRNA in OSCC formation. Tumor metastasis is considered a significant factor in OSCC based on biological processes and potential mechanisms of occurrence. As a result, we carried out pertinent research and discovered that *in vitro* downregulation of hsa_circ_0005991 prevented CAL27 cells from migrating and invading.

In this study, first, we performed high-throughput sequencing and qRT-PCR validation based on collected clinical tissue samples, which means this study has better clinical relevance. Second, we are the first to construct a circRNA-based OSCC-related prognostic model, which shows a good prognostic predictive ability for OSCC patients. Third, the model we constructed can also guide clinicians in choosing appropriate chemotherapy and immunotherapy approaches. However, we also recognize some shortcomings and limitations of this study. First, a circRNA-miRNA-mRNA network was established by bioinformatics analysis. Our study only showed at the *in vitro* level that circRNA can promote the malignant progression of HNSCC cells. Therefore, future studies should validate the current findings in animal models. CircRNAs are found evolutionally conserved in different species, which means some circRNA biomarkers identified in murine modes hold the potential to be translated to clinical application for human beings (Ma et al., 2020). Second, the original dataset for the initial analysis was relatively insufficient, as it was only downloaded from TCGA. While we used various methods to validate the accuracy and validity of our predictive model, more external cohorts are needed in the future to confirm it. Finally, the clinical utility of our risk model, such as its relationship to antitumor drug susceptibility, has not been clinically validated. Therefore, we plan to collect additional clinical samples for RNA-seq to validate our risk model in future experiments and establish more robust clinical links for this new signature.

## Conclusion

In this study, we found that circRNAs were significantly expressed in OSCC compared to normal tissues. We identified four key circRNAs and constructed a circRNA-related ceRNA network associated with OSCC, which will help to elucidate the molecular mechanism of OSCC occurrence and development. In addition, we established an OSCC-related risk model, which may improve OSCC patient survival prediction and reflect immune status, and provide a new reference for personalized treatment.

## Data Availability

The raw sequence data obtained have been deposited at the NCBI in the Sequence Read Archive (SRA) database under bioproject PRJNA904235.
