# Supplementary figures and images for: Circular RNA-related CeRNA network and prognostic signature for patients with oral squamous cell carcinoma
Source: Front Pharmacol. 2022 Dec 1;13:949713. doi: 10.3389/fphar.2022.949713 (PMC9753980; doi:10.3389/fphar.2022.949713)

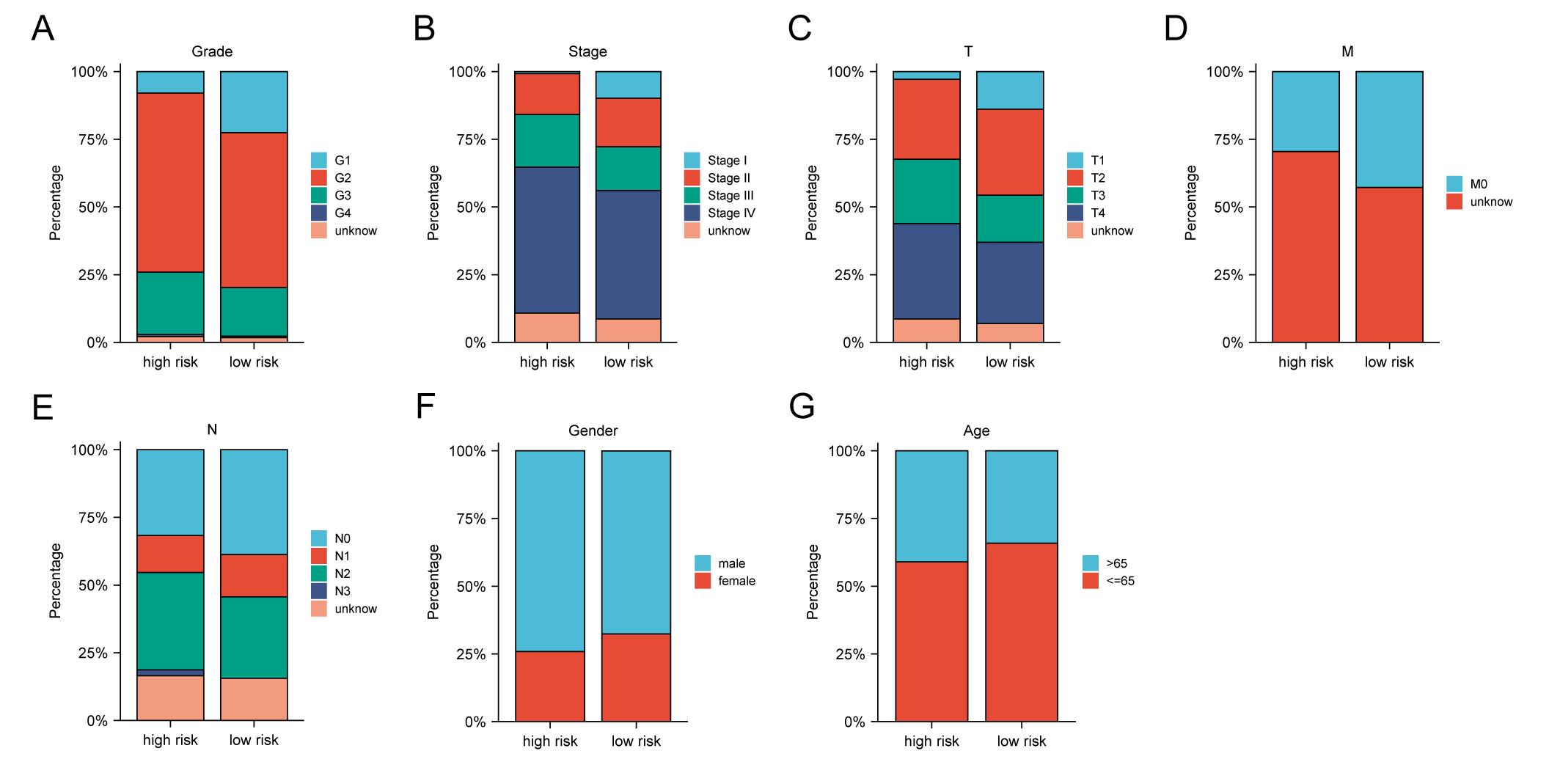

Supplement: Supplementary file 2 [file Image3.TIF]

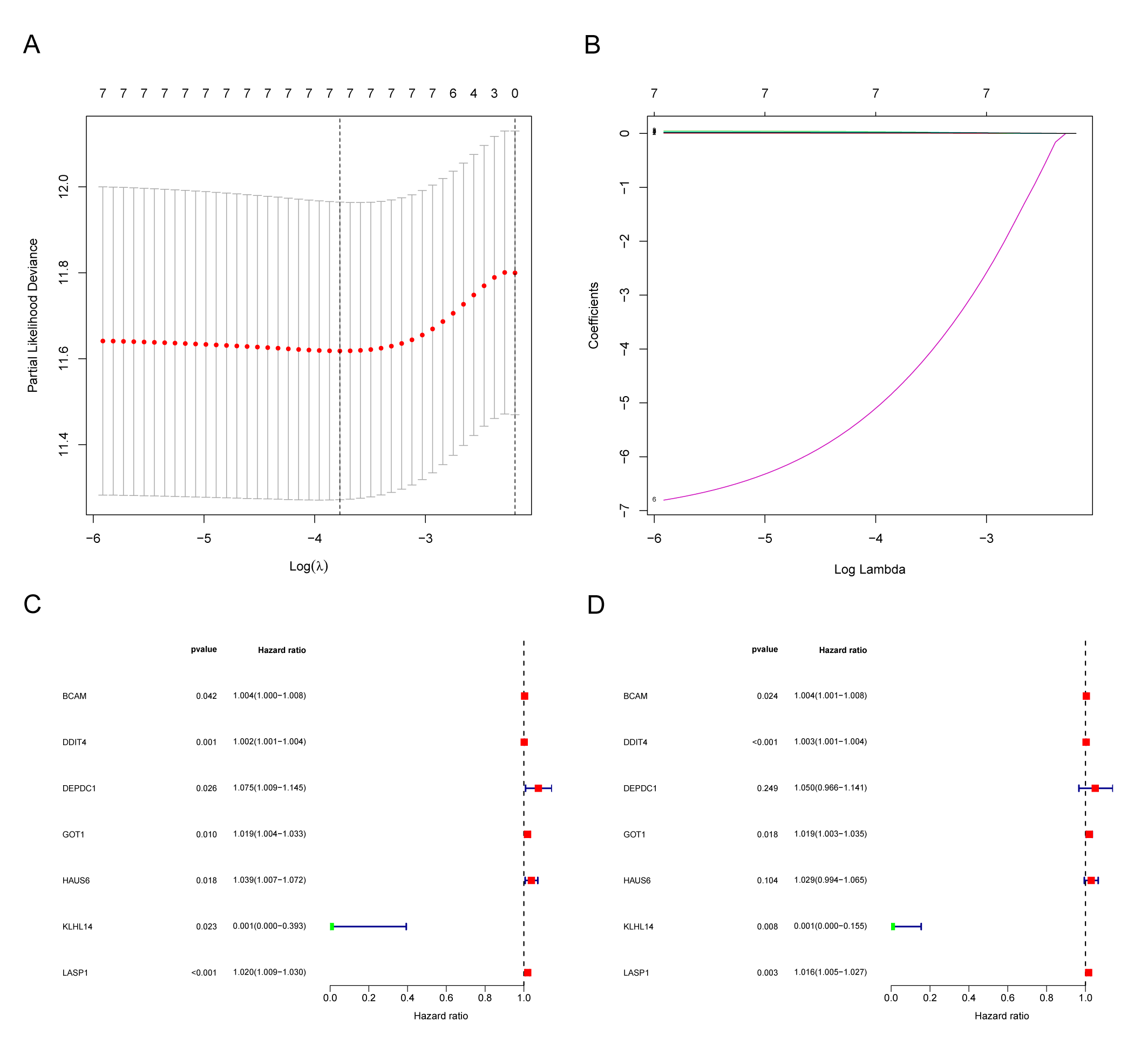

Supplement: Supplementary file 3 [file Image2.TIF]

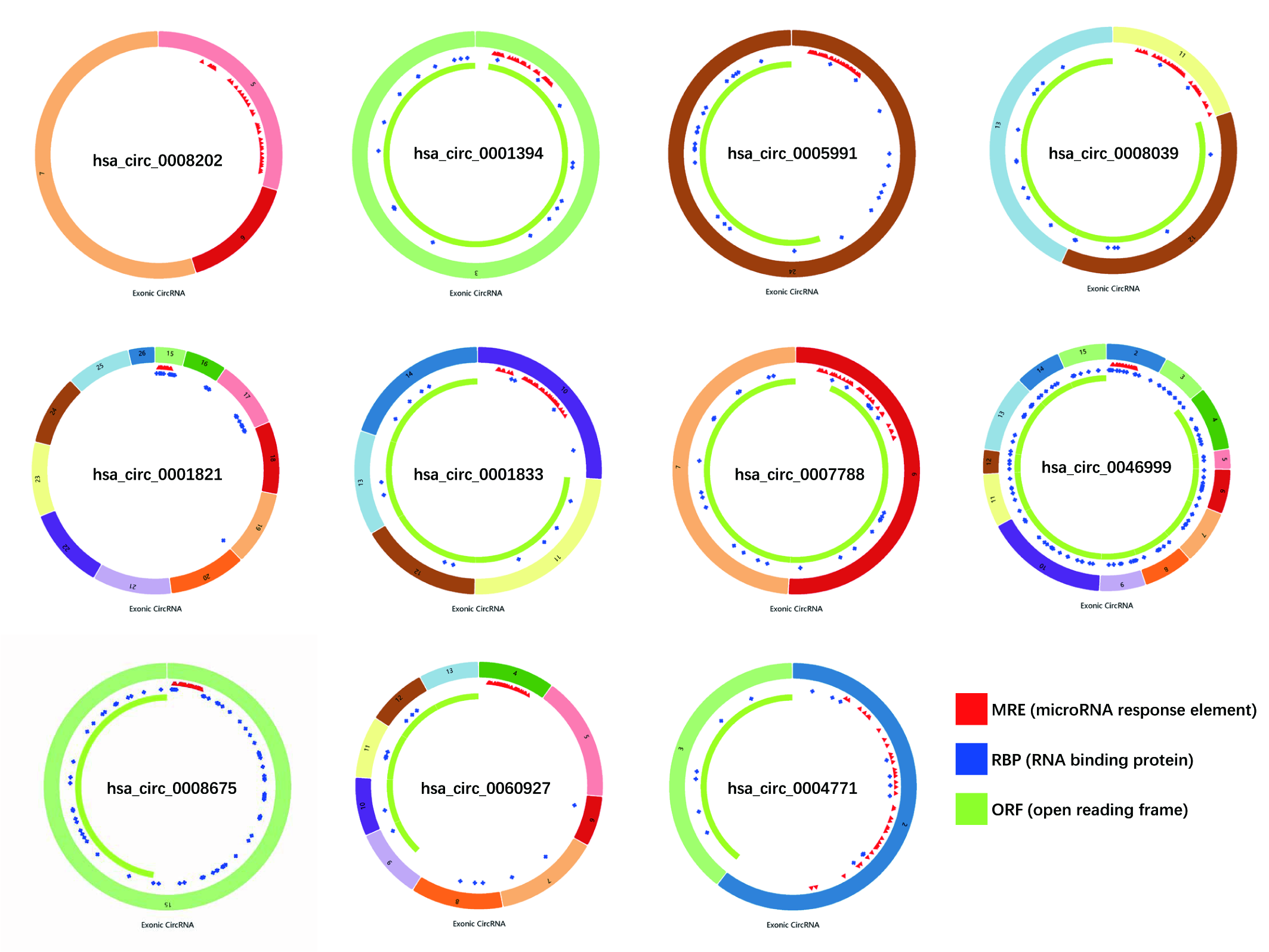

Supplement: Supplementary file 4 [file Image1.TIF]
