# Supplementary material for: Circular RNA-related CeRNA network and prognostic signature for patients with oral squamous cell carcinoma
Source: Front Pharmacol. 2022 Dec 1;13:949713. doi: 10.3389/fphar.2022.949713 (PMC9753980; doi:10.3389/fphar.2022.949713)
Supplement: Supplementary file 5 [file Table2.DOCX]

Supplementary Table 2. Survival analysis of 65 genes in the ceRNA network of OSCC.

| Gene | P-Value |
| --- | --- |
| ADAM10 | 0.0032 |
| HAS2 | 0.0258 |
| ABL2 | 0.0083 |
| TFRC | 0.0079 |
| DEPDC1 | 0.0309 |
| SCN8A | 0.0139 |
| ONECUT2 | 0.0115 |
| IGF1R | 0.0254 |
| PIEZO1 | 0.0234 |
| DDIT4 | 0.0009 |
| LRP12 | 0.0026 |
| CDK6 | 0.0283 |
| TRIM71 | 0.0147 |
| DCBLD2 | 0.0397 |
| MYBL1 | 0.0333 |
| SPOCK1 | 1.50E-05 |
| TGFBR1 | 0.0336 |
| LASP1 | 0.0302 |
| SHC1 | 0.0041 |
| BCAM | 0.0045 |
| MYO10 | 0.0295 |
| ENO2 | 0.0139 |
| RAB3B | 0.0167 |
| CLVS2 | 0.0033 |
| NR3C2 | 0.006 |
| CTNND2 | 0.0277 |
| GFRA1 | 0.0114 |
| RBM24 | 0.0003 |
| KLHL14 | 6.00E-05 |
| DPP10 | 0.0229 |
| SHROOM3 | 0.0333 |
| SLC16A7 | 0.0224 |
| ADAMTS19 | 0.0148 |
| TCEA3 | 0.0007 |
| SASH1 | 0.0048 |
| ETV1 | 0.0213 |
| MPC1 | 0.0333 |
| RAB3C | 0.015 |
| THSD4 | 0.0258 |
| SATB1 | 0.0041 |
| MGAT4A | 0.0228 |
| MYO5B | 0.0088 |
| TMEM47 | 0.0428 |
| ITPR1 | 0.0307 |
| ZNF492 | 0.0098 |
| CRY2 | 0.0406 |
| PLIN1 | 0.0053 |
| TRDN | 0.0016 |
| PRKAA2 | 0.0019 |
| KIT | 0.0394 |
| BEND7 | 0.0033 |
| ZNF43 | 0.0056 |
| GOT1 | 0.0374 |
| FAM91A1 | 0.038 |
| ATP6V1C1 | 0.0317 |
| CDH13 | 0.0035 |
| FBXO5 | 0.0317 |
| SLC16A1 | 0.0307 |
| BCAT1 | 0.0004 |
| ITGA5 | 0.0003 |
| HOXD13 | 0.0194 |
| hsa-miR-548b-3p | 0.0075 |
| hsa-miR-665 | 0.0038 |
| hsa-miR-1276 | 0.0003 |
| hsa-miR-654-3p | 0.0001 |
