# Supplementary material for: Circular RNA-related CeRNA network and prognostic signature for patients with oral squamous cell carcinoma
Source: Front Pharmacol. 2022 Dec 1;13:949713. doi: 10.3389/fphar.2022.949713 (PMC9753980; doi:10.3389/fphar.2022.949713)
Supplement: Supplementary file 7 [file Table4.DOCX]

| gene | HR | HR.95L | HR.95H | pvalue |
| --- | --- | --- | --- | --- |
| BCAM | 1.004081507 | 1.000151124 | 1.008027336 | 0.041802748 |
| DDIT4 | 1.002440889 | 1.000984197 | 1.003899702 | 0.001016912 |
| DEPDC1 | 1.074684177 | 1.008691563 | 1.14499429 | 0.025906466 |
| GOT1 | 1.018836709 | 1.004409187 | 1.03347147 | 0.010330455 |
| HAUS6 | 1.038713474 | 1.006518589 | 1.071938156 | 0.018057935 |
| KLHL14 | 0.001178391 | 3.536628429 | 0.392635417 | 0.022881575 |
| LASP1 | 1.019702172 | 1.009101545 | 1.030414159 | 0.000252945 |
